# As Time Goes by: Anxiety Negatively Affects the Perceived Quality of Life in Patients With Type 2 Diabetes of Long Duration

**DOI:** 10.3389/fpsyg.2019.01779

**Published:** 2019-07-31

**Authors:** Gabriella Martino, Antonino Catalano, Federica Bellone, Giuseppina Tiziana Russo, Carmelo Mario Vicario, Antonino Lasco, Maria Catena Quattropani, Nunziata Morabito

**Affiliations:** ^1^Department of Clinical and Experimental Medicine, University of Messina, Messina, Italy; ^2^Department of Cognitive Sciences, Psychology, Education and Cultural Studies, University of Messina, Messina, Italy

**Keywords:** chronic diseases, anxiety, depression, quality of life, physical component summary, mental component summary, type 2 diabetes

## Abstract

**Introduction:**

Age-related medical conditions are increasing worldwide. Type 2 Diabetes mellitus (T2DM) represents a chronic disease, which affects a large amount of general population, accounting for over 90% of diabetes mellitus (DM) cases.

**Purpose:**

As psychopathological symptoms frequently occur in medical conditions, our study aimed at exploring whether psychological factors and metabolic control may affect health related quality of life (HRQoL).

**Methods:**

Forty five patients with T2DM were consecutively recruited and assessed with a psychodiagnostic battery: Hamilton Anxiety Rating Scale (HAM-A), Beck Depression Inventory II edition (BDI-II) and the 36-Item Short Form Health Survey (SF-36), including indexes Physical and Mental Component Summary (PCS, MCS). Moreover, time since DM diagnosis and glycated hemoglobin (HbA1c) values were detected.

**Results:**

Participants (mean age 65.3 ± 5.9 years) had a mean time since diagnosis of 11.6 ± 6.7 years, and showed a good metabolic control as highlighted by mean HbA1c values 7.1 ± 0.9%. Median HAM-A score [25(20.7–30.6)], represented high prevalence of anxious symptoms. A moderate expression of depressive symptoms was observed [BDI-II score: 13(8.3–21.4)]. A multiple regression analysis, after correcting for age, BMI, HbA1c value and BDI-II score, showed the perceived quality of life relative to PCS was significantly related to both disease duration (β = −0.55, *p* = 0.03, *SE* = 0.25) and HAM-A scores (β = −0.52, *p* = 0.04, *SE* = 0.24). Moreover, both HAM-A (β = −0.67, *p* = 0.01, *SE* = 0.26) and BDI-II (β = −0.48, *p* = 0.02, *SE* = 0.20) scores were independently predictive of MCS. Metabolic control, instead, was not a significant predictor.

**Conclusion:**

Our study suggests a predictive role of both anxiety levels and time since diagnosis in perceived HRQoL in T2DM patients. PCS was associated with anxiety and time since diagnosis and MCS was associated with anxiety and depressive symptoms but not with diabetes duration or metabolic control. These data could be useful to plan T2DM training programs focused on psychological health concerns, possibly leading to a healthy self-management and a better perceived HRQoL, even assisting patients in reducing the negative effect due to the chronicization of T2DM.

## Introduction

Chronical diseases and related outcomes may lead to psychological consequences as they impact both on psychological health and quality of life (Martino et al., 2018a; Kelly et al., 2019). It is known that almost apart from the etiopathological mechanisms, dissimilar age-related chronic diseases may elicit similar psychopathological features, which can even predict morbidity and mortality independently of a comprehensive variety of potential confounders (Kiecolt-Glaser et al., 2002; Lapolla et al., 2012; Martino et al., 2018b; Kelly et al., 2019). Several studies have been conducted exploring both depression and anxiety as predictors of chronic diseases and related outcomes (Atteritano et al., 2013; Catalano et al., 2018). Psychological aspects may also effort people behavior, influencing the management of chronic diseases (Castelnuovo et al., 2015; Van Houtum et al., 2015; Stanton and Hoyt, 2017; Quattropani et al., 2019; Settineri et al., 2019; Vicario et al., 2019). One of the most demanding disease to manage with, due to several variety related concerns, is represented by diabetes mellitus (DM). Differently from autoimmune type 1 DM, Type 2 Diabetes Mellitus (T2DM), also known as adult diabetes, accounts for over 90% of cases and is characterized by a high level of blood glucose due to the body failure to correctly metabolize glucose for the body’s needs, in a context of insulin resistance and relative insulin deficiency. T2DM is a pandemic metabolic disease, with significant morbidity and mortality, estimated to affect at least 285 million people worldwide, and this number will rise to 438 million by the year 2030 (Whiting et al., 2011). It is known that specific features as compliance and adherence are fundamental to adequately manage T2DM long life, reducing the wide related outcomes (Markle-Reid et al., 2018). Accordingly, the emotional distress related to the chronic affection, needs a psychological adaptation process to integrate illness experience into individual’s life context (Whittemore and Dixon, 2008; Whittemore et al., 2010; Van Houtum et al., 2015; Vari et al., 2017; Marchini et al., 2018). Thus, individuals with T2DM try to manage with the stress of living with such chronic illness (Whittemore et al., 2010; Savarese et al., 2018), developing psychological adaptation and adherence through the acceptance of T2DM, including toleration, approval, integration and identification (Lo Coco et al., 2005; Schmitt et al., 2014, 2018; Ebrahimi et al., 2016; Stanton and Hoyt, 2017; Lai et al., 2019). It has been demonstrated that a low psychological adjustment to DM is linked to a worse metabolic control and self-management and lower perceived quality of life (HRQoL) (Misra and Lager, 2008; Smith et al., 2013). Jing et al. (2018) showed that several factors as physical exercise, glucose check, outcomes, time since diagnosis and depression were significantly associated with HRQoL. Individuals with T2DM often report higher levels of depressive symptoms, due to both related complications and disease-management, leading to required life-style changes (Das-Munshi et al., 2007; Bouwman et al., 2010; Van Houtum et al., 2015; Sartorius, 2018). Moreover, external factors as employment and marital status, body mass index (BMI), body image, smoking habits and physical activity could predict depression in T2DM (Bouwman et al., 2010; Guicciardi et al., 2014, 2015; Rosa et al., 2019). Mainly Bouwman et al. (2010) highlighted the association between the higher prevalence of depressive but not anxious symptoms and diabetes. Smith et al. (2013) in a systematic review and meta-analysis found a significant and positive association between diabetes and both anxiety symptoms and anxiety disorder. Anderson et al. (2002) in a meta-analytic review highlighted anxiety disorders are associated with hyperglycemia in T2DM patients. Shinkov et al. (2018) reported the increased prevalence of depression and anxiety among subject with T2DM and metabolic syndrome, underlining depression and anxiety were positively related with age and female gender. Amiri and Behnezhad (2019) confirmed data on the association between anxiety and diabetes, concluding that its outcomes remain controversial and suggesting that diabetes is an important risk factor for anxiety symptoms and therefore that healthy status can prevent anxiety. Nevertheless, the debate about the association between psychopathological factors and chronic medical conditions is still open and there is an increasing interest on the specific role psychological determinant may have on chronic diseases and healthy related quality of life (HRQoL) (Trikkalinou et al., 2017). On the basis of previous studies which explored HRQoL in patients with T2DM (Hasan et al., 2016; Markle-Reid et al., 2018; Nguyen et al., 2018), the aim of our research was to further investigate the relationship between anxious and depressive symptoms, time since T2DM diagnosis and metabolic control on HRQoL, with specific regard to physical and mental component summaries. Our hypothesis was that anxiety may negatively affect HRQoL, with special regard to mental well-being, while diabetes duration and metabolic control may impact on physical well-being.

## Materials and Methods

### Participants

The study was conducted on a group of 45 patients, consecutively recruited at the Outpatients Clinics of the Department of Clinical and Experimental Medicine, University Hospital of Messina, Italy. Patients had a certified diagnosis of T2DM according to the American Diabetes Association criteria (American Diabetes Association., 2018). Inclusion criteria were: age ranging from 55 to 75 years; time since diagnosis of T2DM >5 years; oral treatment with hypoglycemic agent (metformin) at stabilized schedules in the last 12 months, to avoid confounders due to disease severity and related complications, usually associated with both insulin treatment and outcomes; a full screening for diabetic related complications over the last 6 months; Mini-Mental State Examination score (MMSE) >24, to overcome individuals unable to perform or understand the psychological scales. Exclusion criteria were: heart failure with New York Heart Association (NYHA) class >2; moderate and severe respiratory failure; moderate to severe kidney or liver failure; endocrine disorders other than DM (e.g., thyroid or parathyroid disease); severe musculoskeletal disease; cancer; cognitive impairment; neurologic or psychiatric condition or use of neuro-psychotropic drugs.

### Ethics Statement

The study was approved by the Institutional Ethical Committee of the University Hospital “Gaetano Martino,” University of Messina, Messina, Italy. All subjects were deeply informed about the research aim of the study and gave written informed consent in accordance with the Declaration of Helsinki and its later amendments. Participants were evaluated by clinical psychologists in collaboration with physicians. All intervention, including rating scales administration and HbA1c detection, were performed as a part of daily clinical practice assessment of patients. Data were analyzed anonymously.

### Measures

#### Demographical and Medical Data

For each participant data were collected including age, gender, education, smoking habit, employment, and marital status, considered as categorical variables. Medical information comprised data on BMI, T2DM and related complications, time since T2DM diagnosis and metabolic control.

#### Clinical Psychological Evaluation

A gold standard diagnostic interview was performed by researcher in clinical psychology in a confidential setting, to detect patient’s mental status (Fava et al., 2012). It was combined with the use of self-report measures. Particularly, the Hamilton Anxiety Rating Scale (HAM-A) was administered to measure anxiety levels. As known, HAM-A allows to detect both psychological and somatic symptoms, comprising anxious mood; tension; fears; insomnia; intellectual; depressed mood; somatic symptoms; sensory; cardiovascular; respiratory; gastrointestinal; genitourinary; autonomic and observed behavior at interview. Each of 14 items is scored from 0, not present, to 4, severe (Hamilton, 1959). The Beck Depression Inventory-second edition (BDI-II), comprising 21 items, was used to evaluate levels of depression, based on a total score derived from a scale of four points for each proposed item (Beck et al., 1996; Ghisi et al., 2006). The Italian version of the Short Form-36 (SF-36) survey was administered to measure patient’s health perceived HRQoL (Ware and Sherbourne, 1992; Apolone and Mosconi, 1998). It consists of eight multi-items scales assessing *mental health* (reflecting emotional well-being), *role emotional* (reflecting limitations due to emotional health problems), *social functioning*, *vitality*, *general health*, *bodily pain*, *role physical* (reflecting limitations due to physical problems, *physical functioning*. This self-report scale assesses health status via two subscales: Physical Component Summary (PCS) which detects physical well-being and Mental Component Summary (MCS) which captures mental well-being. The SF-36 possible score ranges from 0 to 100 points, with higher scores indicating a better HRQoL. The scoring algorithm for MCS comprises positive weights for vitality, social functioning, role emotional, and emotional well-being scales, and negative weights for the physical functioning, role physical, bodily pain and general health scales. Whereas, the scoring algorithm for PCS covers positive weights for the physical functioning, role physical, bodily pain, general health and vitality scales and negative weights for the social functioning, role emotional, and emotional well-being scales.

#### Clinical Characteristics

Physical evaluation was conducted measuring height and weight, according to standard procedures, and BMI was calculated as weight in kilograms divided by the square of height in meters (Kg/m^2^). Metabolic control was assessed trough the detection of glycated hemoglobin (HbA1c) expressed as per cent value (%), which reflects the mean blood glucose concentration in the last 3 months. T2DM related complications, macro- and microvascular diseases, sensory-motor neuropathy (i.e., hypertension and atherosclerosis nephropathy, retinopathy, glaucoma, and neuropathy) and T2DM related pharmacological treatment were obtained from the patients’ medical records.

### Statistical Analysis

Statistical analyses were performed using MedCalc software (version 10.2.0.0; Mariakerke, 173 Belgium). Kolmogorov-Smirnov test was used to test the normality of distribution of continuous variable. Student’s *t*-test for unpaired observations or Mann-Whitney test were applied. The degree of the association between two variables was analyzed with Spearman’s correlation coefficient. Multiple regression analysis was performed to evaluate the relationship between a dependent variable and one or more independent variables. Values of *p* < 0.05 were considered to indicate statistical significance.

## Results

The recruited 45 participants were predominantly female (70%) and reported a mean age of 65.3 ± 5.9 years. All patients were Caucasian, resident in the South of Italy. The clinical sample characteristics are showed in Table 1.

**TABLE 1:** Demographic and medical characteristics of the study sample.

**Variables**		**Clinical sample (*n* = 45)**
Gender	M	30%
	F	70%
Education	Primary school	38%
	Secondary school	41%
	High school	11%
	Bachelors’s degree	6%
	Ph D or specialization	4%
Employment status	Housewife	44%
	Full time	12%
	Unemployed	0
	Pensioner	44%
Marital status	Never married	6%
	Currently married	67%
	Widowed	15%
	Cohabitant	6%
	Separated or divorced	6%
T2DM time since diagnosis *(yrs)*		11.6 ± 6.7
HbA1c *(%)*		7.1 ± 0.9
T2DM related complications	Micro-vascular diseases	15%
	Macro-vascular diseases	15%
	Macro + Micro	5%
	Sensory motor neuropathy	5%
Age (*yrs)*		65.3 ± 5.9
BMI (*Kg/m^2^)*		29.9 ± 5.2
Current smoking		15%

*Values are expressed as mean ± SD. HbA1c, glycated haemoglobin; T2DM, type 2 diabetes mellitus; BMI, body mass Index.*

Most of the participants had graduated from secondary school (41%) and resulted to be currently married (67%). With regard to the employment status, 44% was represented by pensioner and 44% by housewife, while only 12% was full time. Time since T2DM diagnosis showed a mean value of 11.6 ± 6.7 years. All patients were treated with oral hypoglycemic agents (metformin), showing a good glycemic control and only 40% of them reported T2DM related outcomes, as showed in Table 1.

With regard to the clinical psychological investigation, mainly conducted with the gold standard diagnostic interview, it has been specifically excluded any psychiatric condition. Participants showed moderate depressive symptoms as resulted by median BDI-II score of 13(8.3–21.4), while median HAM-A score [25(20.7–30.6)], considering both somatic and psychic anxiety, was representative of a high prevalence of anxious symptoms in the recruited subjects. Table 2 shows SF-36 scores.

**TABLE 2:** Measurements of health related quality of life (HRQoL) measured by the Short Form-36 (SF-36) questionnaire.

**SF-36 variable**	**Clinical sample median (IQR)**
Mental health	60(51.7−72)
Role emotional	33(0−66)
Social functioning	62(50−62.7)
Vitality	40(34.7−60)
General health	40(35−42.2)
Bodily pain	30(21.8−41.6)
Role physical	50(0−50)
Physical functioning	75(64.7−80.3)
Physical component summary (PCS)	38(33.9−41.1)
Mental component summary (MCS)	36(32−45.2)

**FIGURE 1:**
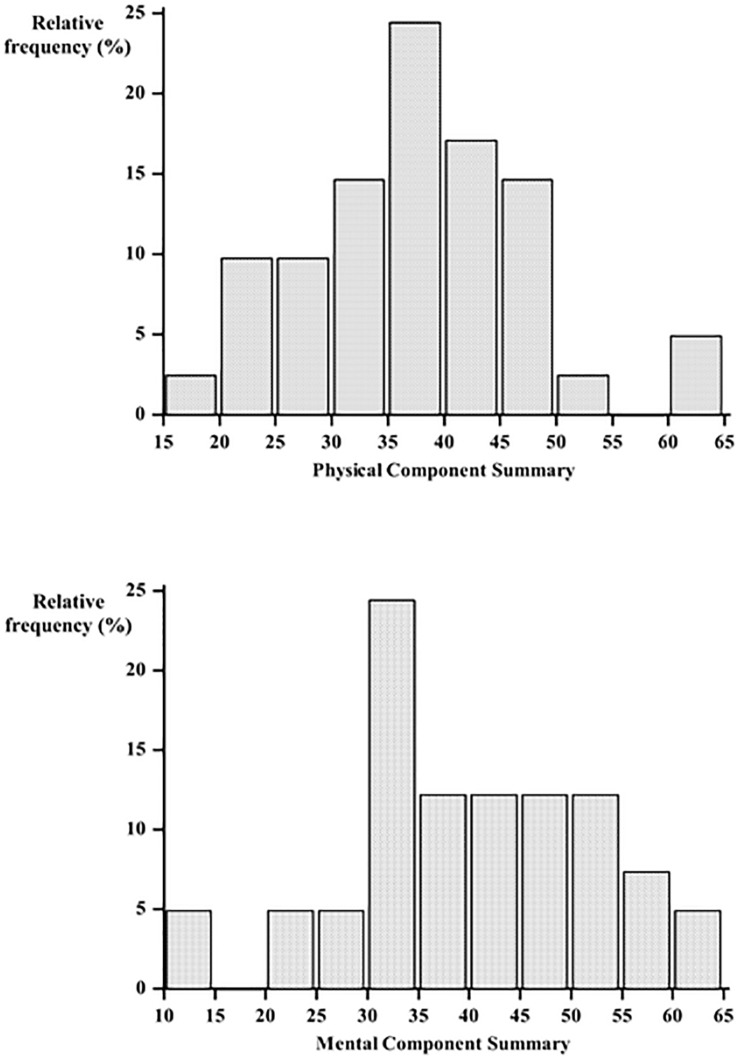
Distribution of Physical Component Summary (PCS) and Mental Component summary (MCS) measured by the Short Form-36 (SF-36).

As observed both PCS and MCS showed a lower perceived HRQoL in the clinical sample (Figure 1).

**FIGURE 2:**
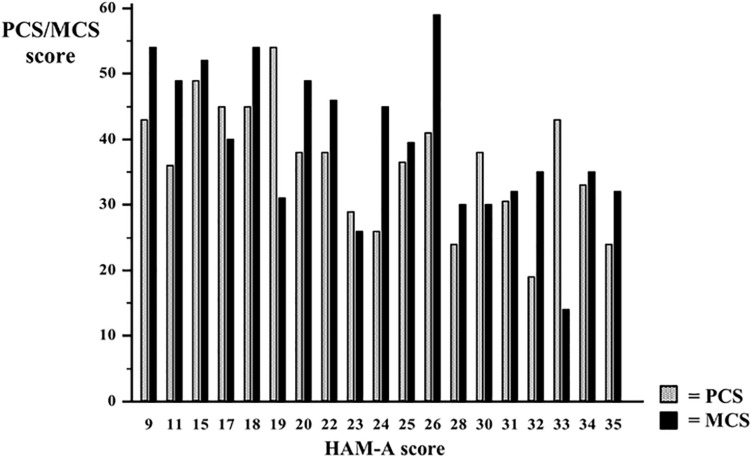
Median Physical Component Summary (PCS) and Mental Component summary (MCS) values according to HAM-A score.

Particularly the lowest scores have been detected in the *role emotional* and *bodily pain* dimensions, while *physical* and *social functioning* showed the highest scores. Figure 1 shows the distribution of *Physical and Mental Component Summary* scores in the clinical sample. Figure 2 shows the median *Physical and Mental Component Summary* scores according to HAM-A values.

All the SF-36 dimensions were inversely and significantly related with anxious and depressive symptoms, except *role physical* and *general health*, as showed in Table 3. Moreover *vitality* was inversely and significantly related to metabolic control. Time since T2DM diagnosis was negatively associated to *physical functioning* and PCS and individuals’ age was inversely related to both *role physical* and *physical functioning* (Table 3).

**TABLE 3:** Correlation coefficients (*r*) between studied variables.

**SF-36**	**Age**	**BMI**	**Time since diagnosis**	**HbA1c**	**HAM-A score**	**BDI-II score**
*Mental health*	–0.15	–0.09	–0.02	0.04	**−0.58**	**−0.48**
*Role emotional*	–0.25	–0.18	0.03	–0.05	**−0.40**	**−0.55**
*Social functioning*	–0.05	**−0.5**	–0.19	–0.12	**−0.48**	**−0.37**
*Vitality*	0.10	–0.17	–0.09	**−0.4**	**−0.65**	**−0.36**
*General health*	0.05	0.12	–0.16	–0.02	**−0.48**	–0.25
*Bodily pain*	–0.02	–0.15	–0.20	–0.16	**−0.65**	**−0.49**
*Role physical*	**−0.32**	–0.30	–0.25	0.05	–0.21	–0.21
*Physical functioning*	**−0.38**	–0.03	**−0.31**	–0.10	**−0.43**	**−0.46**
*Physical component summary*	–0.10	–0.15	**−0.33**	–0.18	**−0.44**	–0.24
*Mental component summary*	–0.07	–0.19	0.01	–0.13	**−0.53**	**−0.45**

*BMI, body mass index; HbA1c, glycated haemoglobin. Statistical significant values of “*r*” (*p* < 0.05) are shown in bold.*

Neither anxiety nor depression were associated with metabolic control and time since diagnosis (*p* > 0.05). Participants with higher education level showed a better but not significant metabolic control in comparison with participants with lower education level [HbA1c 6.9(6.6–7.3) vs. 7.2(6.8–8.8), *p* = 0.08]. Socio-demographic variables, as gender, employment status, marital status and current smoking habit were not significantly associated neither with metabolic control, nor with psychological features.

**FIGURE 3:**
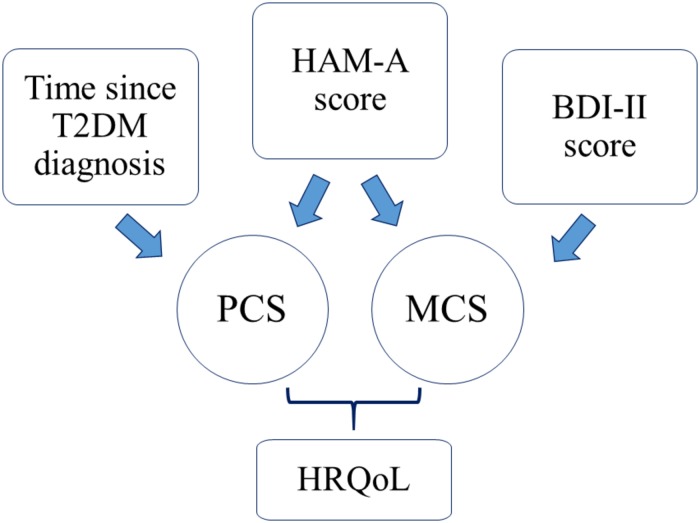
Main determinants of HRQoL in T2DM patients.

A multiple regression analysis, after correcting for age, BMI, HbA1c value and BDI-II score, showed that the PCS was significantly related to both time since diagnosis (β = −0.55, *p* = 0.03, *SE* = 0.25) and HAM-A scores (β = −0.52, *p* = 0.04, *SE* = 0.24). Moreover, after correcting for age, BMI, HbA1c value and T2DM time since diagnosis, both HAM-A (β = −0.67, *p* = 0.01, *SE* = 0.26) and BDI-II (β = −0.48, *p* = 0.02, *SE* = 0.20) scores were independently predictive of MCS (as showed in Figure 3). Metabolic control, instead, was not a significant predictor of HRQoL.

## Discussion

The current study is the first original report aiming to explore the relationship between PCS, MCS, anxious and depressive symptoms, time since diagnosis and metabolic control, in patients with T2DM. Our findings suggest anxiety negatively affects the HRQoL in patients with diabetes of long duration. Anxious symptoms are frequent features in T2DM, which is a chronic progressive condition with physical and psychological concerns (Anderson et al., 2002; Amiri and Behnezhad, 2019). A large population based study showed that people with T2DM had a 20% higher prevalence of life-time diagnosis of anxiety than those without, after adjustment for BMI, educational level, marital and employment status, and current smoking habit (Li et al., 2008). Some explanations about the association between anxiety and diabetes have been proposed (Smith et al., 2013). In fact, it has been reported that a chronic illness such diabetes could lead to an increased risk to develop anxious symptoms, due to both physical symptoms-related worries and disease progression concerns (Goldbacher and Matthews, 2007). It is known that T2DM may provoke distress, as patients are required to self-manage a such chronic disease, frequently measuring blood glucose levels, addressing follow-up with adequate adherence (Marchini et al., 2018). This is also corroborated by Marshall et al. (1997) relatively to psychological distress which may lead to unhealthy behavior impairing compliance and adherence. On the other hand, in a large prospective population-based study, investigating the associations between depression, anxious symptoms and diabetes, individuals with such psychopathological features showed an increased risk of T2DM onset at 10-year follow-up, independently of established risk factors for DM (Engum, 2007; Shinkov et al., 2018). Moreover, the comorbidity of anxiety and T2DM could be due to common factors as pain, complications, un-healthy self-care behaviors, depression and BMI (Engum, 2007; Settineri et al., 2019; Rosa et al., 2019). With regard to the possible pathogenic association between anxiety and T2DM, it has been reported that the development of DM may be due to the stress-induced cortisol release in inflammatory response, even mediated by anxiety (Kiecolt-Glaser et al., 2002; Black, 2003; Seematter et al., 2004; Stellar et al., 2015; Conti et al., 2016). This evidence has been observed in several medical conditions, as cardiovascular and musculoskeletal diseases (Godsland et al., 1998; Catalano et al., 2017).

Particularly, physical outcomes could impair HRQoL as T2DM involves a possible distress related to the long-life self-management, which requires long term follow-up to both ensure metabolic control and to avoid complications. On the other side, anxiety and emotional distress could impair perceived HRQoL, with special reference to the limitations due to emotional problems, which could in turn provoke misadjustment in DM self-care.

Nguyen et al. (2018) found that older age and a longer duration of diabetes were negatively associated with the EQ-5D index, used to evaluate HRQoL. In accordance, we found that time since T2DM diagnosis was strictly related to HRQoL; mainly time since diagnosis was negatively associated to physical functioning and PCS and individuals’ age was inversely related to both role physical and physical functioning.

To properly follow T2DM self-management it could be fundamental patients psychologically to elaborate their chronic illness to find the best adaptive adjustment through self-management strategies. Moreover, such long duration disease may provoke important limitations and impairment of individuals’ life-style. Mainly, T2DM can be also associated to psychopathological symptoms, as occurs in other several chronic conditions, which could impact HRQoL too (Engum, 2007; Crincoli et al., 2016; Catalano et al., 2018). We hypothesized that anxiety, concurring with T2DM features, times since diagnosis and metabolic control, may impair HRQoL and our findings confirmed the significant and negative anxiety association with all SF-36 domains, except role physical, and particularly with mental health, role emotional, social functioning, vitality, general health, bodily pain, physical functioning, PCS and MCS. It could be interesting to comprehend the strictly role of anxiety and T2DM as predictors of lower HRQoL, and mostly on the specific explored dimensions. In our study HRQoL investigation has been carried out including the global SF-36 questionnaire administration, allowing researchers to study the perceived HRQoL regardless of confounders. Mainly, the summarized indexes we investigated, PCS and MCS, have been analyzed in association with time since diagnosis, metabolic control as resulted by HbA1c, and with anxiety levels as resulted by HAM-A scores. We even hypothesized the role of anxious symptoms on MCS and the prevalent impact of metabolic control and diabetes duration on PCS. Our results partially disconfirmed this hypothesis suggesting that anxiety significantly impacts on HRQoL, with regard to both PCS and MCS, but indicated also that diabetes duration, but not metabolic control, impairs on PCS. Moreover, metabolic control does not significantly impair HRQoL dimensions, independently from time since diagnosis, probably due to the recruited clinical sample which was characterized by a good metabolic control, as resulted by HbA1c values almost in therapeutic range. With reference to anxiety levels and its impact on PCS, we could speculate that anxious individuals develop unconscious inhibition and avoiding attitude to perform physical activities, as results from the higher limitations due to physical problems. This theoretical psychodynamic mechanism does not concern depressive symptoms, as demonstrated by the not significant association between depression and PCS, at the linear regression analysis. In our clinical sample depressive symptoms were significantly and inversely associated with mental health, role emotional, social functioning, vitality, bodily pain, physical functioning, and MCS. Furthermore, at a multiple regression analysis, BDI-II score was independently predictive of HRQoL. This data could suggest that even depression, as well as anxiety, significantly and independently impacts on perceived HRQoL in T2DM. Stanton and Hoyt (2017) reported patients with longer time since diagnosis may develop a better adjustment to this chronic disease and to its self-management. Conversely, our findings demonstrated that longer time since diagnosis is predictive of lower HRQoL, with specific reference to PCS and regardless to outcomes. This may be at list in part explained with the underlined psychological features involving emotional stress. Thus, in future researches it could be valuable to plan a clinical psychological intervention strategy, supporting patient to psychologically elaborate and integrate such chronic illness, aimed to a healthier life-style and a better perceived HRQoL, which may also reduce long-life outcomes and complications. This psychological approach, focused on such fundamental issue, could add more qualitative data, even according to a case study approach (Langher et al., 2017). This raise the main role of underlying symbolic and less conscious dynamics, which may include several implications in care relationships (Caputo, 2013; Nazzaro et al., 2017; Tomai et al., 2017).

The strengths of the current study include the multiple regression analysis which allowed us to define the impact of anxiety on HRQoL after multiple adjustment as depressive symptoms, time since diagnosis and metabolic control. Moreover we added to the self-report measures a gold standard as a diagnostic interview, conferring a specific objectivity to the surveys carried out.

Limitations of our study include the cross sectional design, the small sample size and the casual prevalent female gender, which didn’t allow us to observe sub-group analysis (i.e., gender, exercises) which is, however, what we aim to perform as our main perspective research. Other limitations are: the oral anti-diabetic treatment represented by metformin, which conferred on the other side homogeneity to the sample; the use of BDI-II even if there are many depression inventories validated in diabetes cohorts, which is due to a general standardized evaluation for outpatients referring to our Department for chronic diseases.

## Conclusion

Our study suggests a predictive role of both anxiety levels and time since diagnosis in HRQoL in a chronic disease such as T2DM. Our findings highlighted that higher PCS levels, expression of a better HRQoL with regard to physical component, were associated to lower anxiety levels and less time passed since T2DM diagnosis. Moreover, higher MCS levels, expression of a better HRQoL with regard to mental component, were associated to lower anxious and depressive symptoms but not with diabetes duration or metabolic control. These data could be useful to plan T2DM psychological intervention focused on psychological health concerns, leading to a healthy self-management and a better HRQoL, even assisting patients in reducing the psychological and physic outcomes due to the T2DM long duration.

## Data Availability

The raw data supporting the conclusions of this manuscript will be made available by the authors, without undue reservation, to any qualified researcher.

## Author Contributions

GM made significant contribution to design the research study, to draft the manuscript and to provide the interpretation of data. AC performed the statistical analysis by providing the interpretation of data and provided significant contribution to draft part of the manuscript. FB, GR, and AL provided substantial contribution in drafting part of the manuscript. CV and MQ revised the article critically. NM gave the final approval of the version of the manuscript to be submitted.

## Conflict of Interest Statement

The authors declare that the research was conducted in the absence of any commercial or financial relationships that could be construed as a potential conflict of interest.
